# Characterization of a Novel CaCO_3_-Forming Alkali-Tolerant *Rhodococcus erythreus* S26 as a Filling Agent for Repairing Concrete Cracks

**DOI:** 10.3390/molecules26102967

**Published:** 2021-05-17

**Authors:** Seunghoon Choi, Sungjin Park, Minjoo Park, Yerin Kim, Kwang Min Lee, O-Mi Lee, Hong-Joo Son

**Affiliations:** 1 Department of Life Science and Environmental Biochemistry, Life and Industry Convergence Research Institute, Pusan National University, Miryang 50463, Korea; cookie1232@pusan.ac.kr (S.C.); shcv2966@pusan.ac.kr (S.P.); alswn940809@pusan.ac.kr (M.P.); kimyl0508@pusan.ac.kr (Y.K.); leekm@pusan.ac.kr (K.M.L.); 2 Avian Disease Division, Animal and Plant Quarantine Agency, Gimcheon 39660, Korea; lomi78@korea.kr

**Keywords:** calcium carbonate, cementitious materials, concrete, repair, ureolysis

## Abstract

Biomineralization, a well-known natural phenomenon associated with various microbial species, is being studied to protect and strengthen building materials such as concrete. We characterized *Rhodococcus erythreus* S26, a novel urease-producing bacterium exhibiting CaCO_3_-forming activity, and investigated its ability in repairing concrete cracks for the development of environment-friendly sealants. Strain S26 grown in solid medium formed spherical and polygonal CaCO_3_ crystals. The S26 cells grown in a urea-containing liquid medium caused culture fluid alkalinization and increased CaCO_3_ levels, indicating that ureolysis was responsible for CaCO_3_ formation. Urease activity and CaCO_3_ formation increased with incubation time, reaching a maximum of 2054 U/min/mL and 3.83 g/L, respectively, at day four. The maximum CaCO_3_ formation was achieved when calcium lactate was used as the calcium source, followed by calcium gluconate. Although cell growth was observed after the induction period at pH 10.5, strain S26 could grow at a wide range of pH 4–10.5, showing its high alkali tolerance. FESEM showed rhombohedral crystals of 20–60 µm in size. EDX analysis indicated the presence of calcium, carbon, and oxygen in the crystals. XRD confirmed these crystals as CaCO_3_ containing calcite and vaterite. Furthermore, *R. erythreus* S26 successfully repaired the artificially induced large cracks of 0.4–0.6 mm width.

## 1. Introduction

Calcium carbonate (CaCO_3_) formation is a representative example of bacterial mineralization occurring because of the involvement of various bacterial genera in a wide range of natural environments; this process is known as bacterially induced CaCO_3_ precipitation (BICP) [1]. Recent studies on BICP cover a variety of fields with important implications for geobiotechnology, civil engineering, and the environment, such as biocement, concrete reinforcement, soil stabilization, carbon dioxide sequestration, wastewater treatment, heavy metal bioremediation, and oil recovery [2,3]. The most promising among them is the use of BICP to repair damaged concrete structures and historically important stone cultural heritages [4].

Various processes, such as physicochemical weathering, biological corrosion, ground subsidence, and human activity, cause cracks in concrete and stone structures. If not treated properly and quickly, cracks will expand further, eventually requiring costly repairs. Currently, resin mortar and epoxy are used to repair cracks, but they react with nitrogen oxides to create ozone, a highly toxic air pollutant which is harmful to humans and the environment [5]. Therefore, from the viewpoint of eco-friendliness and cost reduction, new cementitious materials for repairing structural cracks such as BICP, which is known as the self-healing technique, have gained attention. Self-healing materials refer to substances that can restore themselves to their original state. In particular, bacterially induced self-healing is described as the process of autonomously sealing cracks generated on the surface of concrete structures by chemical changes (i.e., CaCO_3_ formation) owing to the metabolic activity of bacteria [5,6].

Several processes have been found in which CaCO_3_ is formed by bacteria, including urea hydrolysis, denitrification, sulfate reduction, photosynthesis, and methane oxidation [6,7]. Among them, urea hydrolysis is the most studied and widely applied because of its excellent CaCO_3_ formation efficiency [8,9]. To date, most investigations on BICP by ureolytic bacteria have been conducted using the urease-producing strain of *Sporosarcina pasteurii* [10,11,12]. This strain can increase the pH by hydrolyzing urea to produce ammonium and bicarbonate ions, thereby accelerating CaCO_3_ formation [8]. However, because concrete is a harsh environment with a high pH, crack repair requires microorganisms that can survive and grow in such an environment. Therefore, the isolation and characterization of other extreme microorganisms are important tasks with regard to securing new biological resources for broader industrial applications.

As part of a project on the isolation of extreme microorganisms, we previously isolated a new strain capable of forming CaCO_3_ from alkaline soil (pH 9) [13]. In this study, the BICP characteristics of the isolated strain and the morphological characteristics of the formed CaCO_3_ were investigated. Furthermore, the rapid crack-repairing ability of this strain was confirmed.

## 2. Results and Discussion

### 2.1. Urease Activities in Solid and Liquid Media

**Figure 1:**
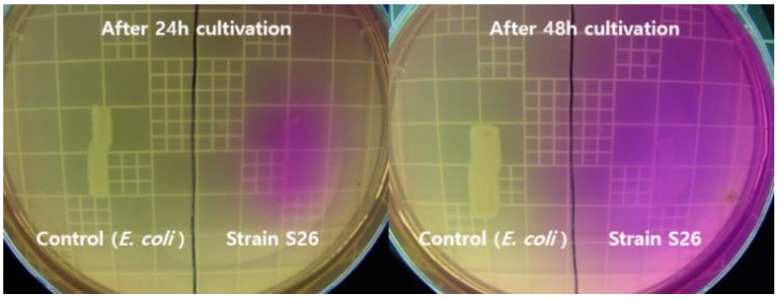
Photographs showing urease activity of *R. erythreus* S26 and *E. coli* (urease-negative strain) on a urea agar base plate.

*Rhodococcus erythreus* S26 was inoculated on a urea agar base plate to investigate whether the color change of the medium was caused by a pH increase due to urease activity. As shown in Figure 1, the color of the medium changed from yellow to pink at 24 h, after which the color change of the medium continued to expand, indicating a quick and widespread increase in pH was apparent in strain S26. In contrast, the urease-negative bacterium, *E. coli*, did not change the color of the medium.

**Figure 2:**
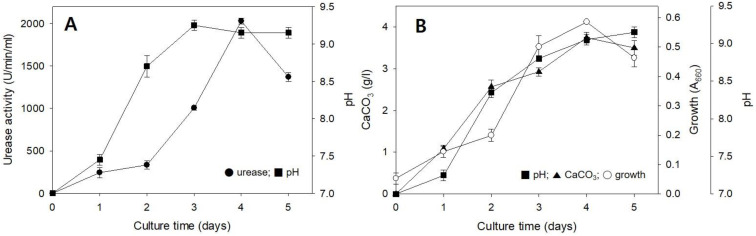
Urease activity (**A**) and CaCO_3_ formation (**B**) of *R. erythreus* S26 in a liquid medium containing CaCl_2_. Error bars (±SDs) are shown when larger than the symbol.

Figure 2A shows the change in urease activity according to the incubation time in NB-urea broth. After urease of 285 U/min/mL was produced at 24 h, it increased proportionally over time, showing maximum activity (2054 U/min/mL) at 4 d. Culture pH increased from 7.0 to 9.1 after 3 d of incubation.

### 2.2. CaCO_3_ Formation in Solid and Liquid Media

**Figure 3:**
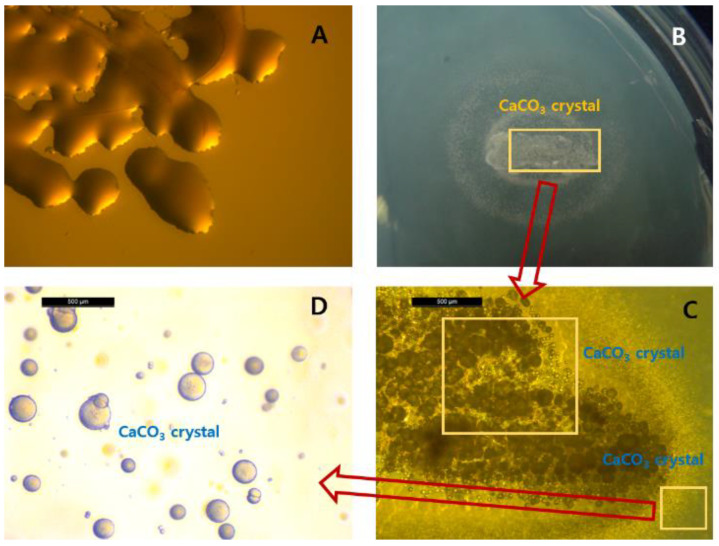
Photographs of crystals formed by *R. erythreus* S26 on a urea-CaCl_2_ agar plate (**A**), *E. coli* (urease-negative strain) (**B**–**D**), *R. erythreus* S26.

CaCO_3_ formation by strain S26 was investigated using a urea-CaCl_2_ plate. As shown in Figure 3, CaCO_3_ crystals of various sizes were observed in the colony and the surrounding medium. In particular, glossy crystals were densely distributed covering the colony surface. Most of these crystals were spherical; however, some polygons were also observed. Only spherical crystals were observed in the medium without cell growth around the colonies. No mineral crystals were observed in urease-negative *E. coli*. The shape and size of CaCO_3_ crystals depend on the molecular properties of exopolysaccharides secreted by bacterial cells, as well as on the colony form [14]. Furthermore, spherical CaCO_3_ crystals, vaterites, are primarily formed in solid medium containing agar [15].

Figure 2B shows typical time courses for pH, cell growth, and CaCO_3_ formation in the urea-CaCl_2_ broth. The CaCO_3_ was formed (3.83 g/L) at 4 d of cultivation when the cell growth reached the late logarithmic growth phase. Cell growth showed the same tendency as observed for CaCO_3_ formation. The pH of the culture gradually changed from neutral to alkaline due to urea degradation and showed a pattern similar to that of NB-urea broth. In addition, CaCO_3_ formation was found to be proportional to urease activity (Figure 2A). From the data shown in Figure 2, the CaCO_3_ formation process can be explained as follows. In the urea-CaCl_2_ medium, bacterial urease broke urea into ammonium ions, thereby increasing the pH of the medium. The negatively charged site of the bacterial cell wall was a nucleation site where calcium ions bound, and carbonate ions bound to calcium ions sequentially, forming CaCO_3_ crystals [16].

CO(NH_2_)_2_ + 2H_2_O → 2NH_4_^+^ + CO_3_^2−^ (1)

Ca^2+^ + Cell → Cell-Ca^2+^ (2)

Cell-Ca^2+^ + CO_3_^2−^ → Cell-CaCO_3_ (3)

### 2.3. Influence of Calcium Source on CaCO_3_ Formation

**Figure 4:**
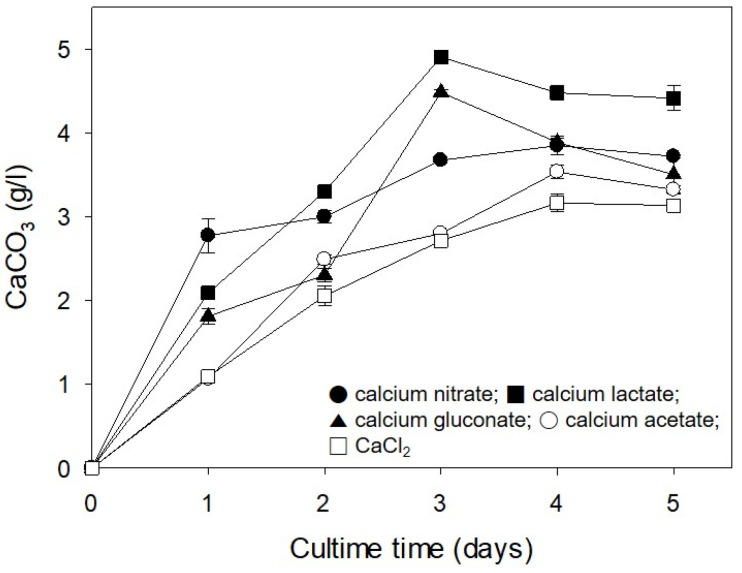
Influence of the calcium source on CaCO_3_ formation by *R. erythreus* S26. Error bars (±SDs) are shown when larger than the symbol.

The CaCO_3_ formation by microorganisms is greatly influenced by the type of calcium salt [17,18]. Therefore, CaCO_3_ formation by strain S26 was investigated using different calcium sources. In all experimental groups, the amount of CaCO_3_ formed increased in proportion to the incubation time, with the maximum values being observed at 3–4 days. CaCO_3_ formation was in the following order: calcium lactate, calcium gluconate, calcium nitrate, CaCl_2_ (Figure 4).

One of the main issues in applying CaCO_3_-forming microorganisms to concrete crack repair is in exploring the biochemical process of carbonate mineral formation. This process involves converting the soluble calcium source into insoluble CaCO_3_; therefore, the influence of the calcium source type is important. Most previous studies have used CaCl_2_ as the calcium source [17,18,19]. Seifan et al. [20] examined the effects of various calcium sources on CaCO_3_ formation using *Bacillus* species and found that CaCl_2_ was the best source of calcium for the formation of CaCO_3_. Lee [21] reported that the maximum CaCO_3_ yield by *Bacillus amyloliquefaciens* CMB01 was achieved using calcium acetate. Hence, in the present study, we suggest that the optimal calcium source for CaCO_3_ formation varies depending on the microorganism used.

### 2.4. Influence of pH on Cell Growth

**Figure 5:**
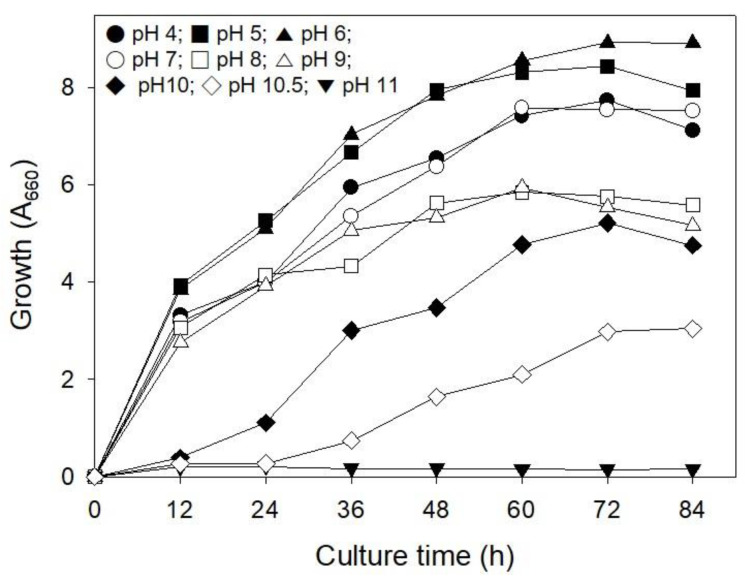
Influence of culture pH on cell growth of *R. erythreus* S26. Error bars (±SDs) are shown when larger than the symbol.

Many microorganisms cannot grow in concrete, which is a highly alkaline environment. To repair cracks in concrete using microorganisms, they must survive and grow in extreme environments [22]. Therefore, the effect of pH on the growth of strain S26 was investigated. As shown in Figure 5, strain S26 showed high growth at pH 4–7, followed by pH 8–10. The cells were able to grow after a lag period of 24 h at pH 10.5, but not at pH 11. Additionally, concrete is strongly alkaline with a pH 11, but the pH decreases to pH 8–10 when moisture penetrates inside it through the cracks created [22]. Considering this, strain S26 can form CaCO_3_ after the adaptation period in strongly alkaline concrete and has the potential for in situ applications.

### 2.5. Structural Characterization of CaCO_3_ Crystals

**Figure 6:**
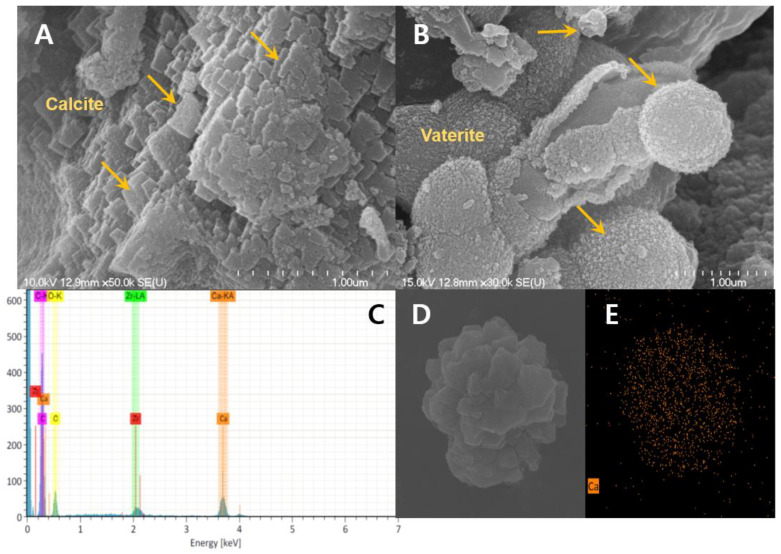
FESEM images (**A**,**B**), elemental mapping (**C**,**D**), and EDX spectrum (**E**) of CaCO_3_ crystals formed by *R. erythreus* S26.

Morphology of CaCO_3_ formed in the urea-calcium lactate broth was investigated using FESEM. FESEM images showed that the formed CaCO_3_ crystals were mostly rectangular or rhombohedral with an irregular structure, with a size of 0.15–0.25 µm (Figure 6A). A few spherical crystals with particle sizes of 0.35–1.1 µm were also observed (Figure 6B). The elemental composition of the CaCO_3_ crystals was investigated using EDX and elemental mapping. The EDX spectrum showed that the sample contained Ca (25.2 atomic %), C (16.5% atomic %), and O (45.0 atomic %), indicating the presence of CaCO_3_ crystals (Figure 6C). Further elemental mapping confirmed that the crystals consisted of CaCO_3_ (Figure 6D,E).

**Figure 7:**
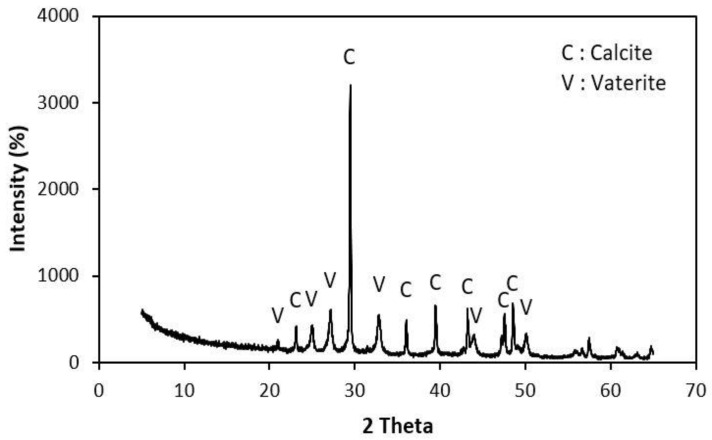
XRD pattern of CaCO_3_ crystals formed by *R. erythreus* S26.

As shown in Figure 7, XRD analysis of the crystals showed that two CaCO_3_ polymorphs were formed during the growth in urea-calcium lactate broth; the primary crystal component was rhombohedral calcite, but a small amount of spherical vaterite was also detected, which confirmed the results of FESEM imaging. Thermodynamically, calcite is the most stable CaCO_3_ polymorph and is a major product of many microbial CaCO_3_ formation processes [23]. In contrast, *Proteus mirabilis* [24] and *Delia halophila* [25] form vaterite and aragonite, respectively. Park et al. [26] reported that proteins or minerals produced during metabolism by CaCO_3_-forming microorganisms were adsorbed on specific crystallographic planes, thereby changing the shape of the crystals. These results suggest that the morphological specificity of the crystals formed is due to differences in the bacterial genera.

### 2.6. Concrete Crack-Repairing Effect

**Figure 8:**
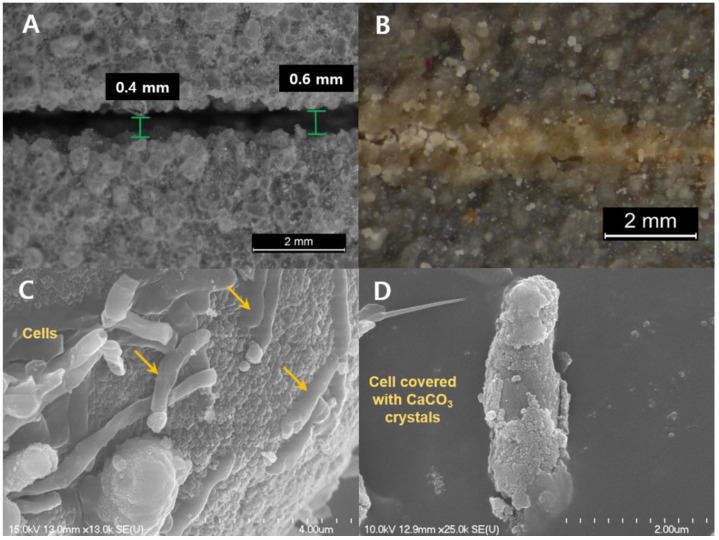
Repair of a concrete crack by *R. erythreus* S26-induced CaCO_3_ before (**A**) and after (**B**) incubation. B is a photograph showing that the crack was sealed when the cells were cultured for 6 d. (**C**,**D**) are FESEM images showing CaCO_3_ crystals and CaCO_3_-forming cells in the crushed sample after crack repairing.

In order to repair concrete cracks using microbial CaCO_3_ formation, artificial cracks (0.4–0.6 mm) were made and cells were inoculated. As shown in Figure 8A,B, CaCO_3_ precipitates were found in cracks treated with the S26 strain; these cracks were gradually repaired over time and sealed tightly after 6 d. To confirm the microstructure of CaCO_3_ formed in the cracks, the repaired sample was crushed by hand, and then small pieces of CaCO_3_ were observed with FESEM. The formed CaCO_3_ crystal was a compact layered structure composed of polygonal and spherical particles, and rod-shaped cells were observed on its surface (Figure 8C). Some cells appeared to be fossilized by being covered with CaCO_3_ crystals (Figure 8D). De Muynck et al. [27] reported that this dense structure increased the strength of the concrete by giving it stronger adhesion and more tightness. Furthermore, the presence of cells is evidence that strain S26 was directly involved in crack repair, indicating that bacteria act as nucleation sites in the calcium carbonation process. On the other hand, the treatment of a wide crack with CaCO_3_-forming bacteria does not provide the nucleation site required for CaCO_3_ crystal formation because the cells flow down without attaching to the inner surface of the crack [27]. For example, unlike the results of this study, Park et al. [28] reported that there was no repair effect when *Raoultella ornithinolytica*, *Stenotrophomonas maltophilia*, and *Bacillus thuringiensis* were tested on cracks with widths of 0.22–0.57 mm. Exceptionally, it was reported that *Bacillus sphaericus* LMG25557 completely repaired cracks up to 0.97 mm wide in 21 days [28]. These results indicate that the repairing effect of concrete cracks depend on the microbial species, and strain S26 can be applied to relatively large cracks. Meanwhile, van Titelboom et al. [29] reported that the crack-repairing effect was increased by using resin as a binder that increased the microbial adhesion to cracks with large widths.

## 3. Materials and Methods

### 3.1. Bacterial Strain and Culture Conditions

*R. erythreus* S26 isolated from alkaline soil in Korea was used in this study [13]. It can form CaCO_3_ in a urea- and CaCl_2_-containing medium. Nutrient broth was used for the preservation and pre-culture of the strain. For pre-culture, a 250 mL Erlenmeyer flask containing 50 mL of nutrient broth was inoculated with a colony from a culture plate, followed by incubation at 30 °C and 200 rpm for 18 h. Unless otherwise noted, 2% (*v*/*v*) of the pre-culture was inoculated into the main medium and incubated under the conditions mentioned above.

### 3.2. Urease Activities in Solid and Liquid Media

The S26 strain was inoculated on a urea agar base plate (0.1% gelatin, 0.1% glucose, 0.5% NaCl, 0.2% K_2_HPO_4_, 2% urea, and 0.0012% phenol red, pH 7.0) and incubated at 30 °C for 24–48 h. This medium also contained phenol red, which indicates pH change associated with ammonia production due to urea hydrolysis. A color change from orange to pink indicated urea hydrolysis. A negative control was prepared using *Escherichia coli* KCCM 40880, a known non-ureolytic bacterium.

In addition, strain S26 was inoculated into NB-urea broth (0.3% nutrient broth and 2% urea, pH 8.0) and incubated at 30 °C, and time-dependent urease activity was investigated. Urease activity was determined using the method described by Natarajan [30] with some modifications. Briefly, 250 µL of sample was added to a previously mixed 250 µL of 0.1 M potassium phosphate buffer (pH 8.0) and 500 µL of 0.1 M urea solution. The mixture was incubated at 37 °C for 5 min, after which 500 µL of phenol nitroprusside solution was added, followed by 500 µL of alkaline hypochlorite solution. The mixture was then incubated at 37 °C for 25 min, followed by absorbance measurement at 626 nm, using ammonium chloride (0–100 μM) as the standard. One unit of urease activity is defined as the amount of enzyme hydrolyzing 1 μmol urea/min/mL.

### 3.3. CaCO_3_ Formations in Solid and Liquid Media

To investigate the formation of CaCO_3_ by strain S26, cells were inoculated on a urea-CaCl_2_ plate (0.3% nutrient broth, 2% urea, 0.212% NaHCO_3_, 1% NH_4_Cl, 25 mM CaCl_2_, and 1.5% agar, pH 7) and incubated at 30 °C. CaCO_3_ crystals formed around the colonies were observed using a stereoscopic microscope (Laica S6 D, Leica Microsystems, Wetzlar, Germany).

To quantify CaCO_3_ formation, cells were inoculated into urea-CaCl_2_ broth and incubated at 30 °C and 200 rpm, and the amount of CaCO_3_ was measured over time. The culture was centrifuged at 500× *g* for 3 min to remove the supernatant, and the precipitated CaCO_3_ was collected. CaCO_3_ was washed with distilled water to remove residual cells and culture components and was dried at 105 °C for 2 h to measure the dry weight [31].

### 3.4. Influence of Calcium Source on CaCO_3_ Formation

Urea-CaCl_2_ broth was used as a basic medium to study the effect of various calcium salts on the formation of CaCO_3_ by strain S26. Calcium sources (calcium acetate, calcium gluconate, calcium lactate, calcium nitrate, and CaCl_2_) at 25 mM were separately added to the medium. Cultivation was performed at 30 °C and 200 rpm, and CaCO_3_ formation was measured at each incubation time.

### 3.5. Influence of pH on Cell Growth

To investigate the pH tolerance of strain S26, cells were inoculated into tryptic soy broth adjusted to pH 4–11, respectively, and incubated at 30 °C and 200 rpm. Cell growth was measured at 660 nm in a time-dependent manner.

### 3.6. Crystal Analysis

The morphology of the CaCO_3_ crystals was studied using field emission scanning electron microscopy (FESEM) (Carl Zeiss, SUPRA 40VP, Oberkochen, Germany). Prior to analysis, the dried CaCO_3_ crystals were mounted on specimen stubs with double adhesive tape, sputter-coated with platinum, and examined at 40 kV. Elemental analysis was then conducted using an energy dispersive X-ray (EDX) detector connected to the FESEM. X-ray diffraction (XRD) analysis of the CaCO_3_ crystals was performed using a Panalytical diffractometer (Empyrean series 2, Almelo, Netherlands) at 40 kV and 30 mA using CuKα radiation (λ = 1.54 Å). Data were collected in the 2θ range of 20°–80° at a scan rate of 10°/min.

### 3.7. Repairing Test of Concrete Crack

A concrete paste was prepared by mixing cement (ordinary Portland cement type 1), sand, and water at a ratio of 6:3:4 (*w/w*), and the pH of the finished concrete paste was 10.6. Artificial cracks were induced by casting concrete paste on a Petri dish, inserting a 150 μm thick polyester film up to a depth of 5 mm, and placing it at room temperature for 1 d. After removing the film, the cracked concrete was cured for 20 d in tap water.

The crack repair effect of strain S26 was investigated using a cured concrete specimen. Strain S26 was cultured in nutrient broth (30 °C, 200 rpm, 24 h), and then centrifuged at 12,000× *g* for 5 min to collect the cells. The cell suspension (100 µL) diluted to A_660_ = 1 in urea-calcium lactate medium was injected into the crack every 12 h (repeated for 3 d), and the crack repair was observed using the stereoscopic microscope while incubating at 30 °C.

## 4. Conclusions

In the present study, we described an environment-friendly and efficient microbiological method for sealing cracks using *R. erythreus* S26 as a concrete healing agent. This method does not require the use of hazardous chemicals to repair concrete cracks. Strain S26 was able to produce the urease required for CaCO_3_ formation. When calcium lactate was supplied as a calcium source, the CaCO_3_ formation was the highest, and the formed CaCO_3_ crystals were a mixture of calcite and vaterite. In addition, strain S26 was able to grow in a strongly alkaline environment with a pH of 10.5. These results imply that *R. erythreus* S26 is not only useful for in situ repair of concrete cracks, but also to improve the compressive strength of mortar. To explore these possibilities, detailed studies on the reinforcement and durability of concrete are currently being conducted. In addition, research on the development of an economical medium capable of maximizing CaCO_3_ formation for the efficient repair of wider cracks within a shorter time should be carried out.

## Data Availability

The data presented in this study are available in the article.

## Sample Availability

Samples of the compounds are not available from the authors.
